# Scapular force: Couple ratios in healthy shoulders – An observational study reflecting typical values

**DOI:** 10.4102/sajp.v78i1.1619

**Published:** 2022-07-25

**Authors:** Sonia Briel, Benita Olivier, Witness Mudzi

**Affiliations:** 1 Department of Physiotherapy, Faculty of Health Sciences, University of the Witwatersrand, Johannesburg, South Africa

**Keywords:** scapular stability, force couple ratios, stabilising muscles, shoulder

## Abstract

**Background:**

Scapular stability is primarily maintained through the action of the scapular stabilisers and not through bony stability. The values of the force couple ratios of the scapular stabilisers remain largely unknown.

**Objectives:**

To determine typical scapular force couple strength ratios in the pain-free shoulders of healthy female and male participants.

**Methods:**

This was a quantitative cross-sectional study. The muscle strength of the serratus anterior upper and lower fibres, the upper, middle and lower trapezius and the rhomboids (in both shoulders) were determined in kilogram force (kgf) using a handheld dynamometer. The ratios of the force couples of the scapulae of both shoulders of the participants were calculated. Participants (both female and male) with healthy shoulders were recruited from the general public (a local university, schools, church groups and sport clubs). We mainly utilised descriptive analysis. Statistical significance was set at 5%.

**Results:**

Force couple ratios were as follows (means, with SD). Dominant arm in women: upper trapezius:lower trapezius 3.63 (0.97); serratus anterior lower fibres:lower trapezius = 1.97 (0.27); middle trapezius:serratus anterior upper fibres = 0.40 (0.10); serratus anterior lower fibres:rhomboids = 1.41 (0.21); lower trapezius:rhomboids = 0.74 (0.17). Dominant arm in men: upper trapezius:lower trapezius = 2.70 (0.72); serratus anterior lower fibres:lower trapezius = 2.15 (0.45); middle trapezius:serratus anterior upper fibres = 0.47 (0.12); serratus anterior lower fibres:rhomboids = 1.40 (0.31) and lower trapezius:rhomboids = 0.17 (0.6).

**Conclusion:**

Specific force couple strength ratios were determined, between and within the nondominant and the dominant arms of the shoulders of healthy women and men.

**Clinical implications:**

Scapular stability is mainly maintained through the optimal force couple balance of the scapular stabilisers.

## Introduction

The poor anchoring of the scapula by bony attachments results in the dynamic and static stability of the scapula being largely dependent on the balanced actions of the scapular musculature (Du et al. 2020; Kibler & Sciascia 2010; Kibler et al. 2013; Michener et al. 2016; Sahrmann 2001). Historically, it has generally been accepted that proper dynamic scapular stability is provided through the combined actions of the force couples of the serratus anterior and all the parts (upper, middle and lower) of the trapezius (Bagg & Forrest 1988; Inman et al. 1944). More recent studies conducted on scapular stability echo these findings (Kibler 1998a; Michener et al. 2016). Anatomically, the individual parts of the trapezius are one muscle, as are the individual parts of the serratus anterior muscle (Johnson et al. 1994; Smith et al. 2003). However, biomechanically, the individual parts (upper, middle and lower trapezius and the upper and lower serratus anterior) display distinctive functions (Ekstrom, Bifulco & Lopau 2004; Fey et al. 2007; Wiedenbauer & Mortenson 1952).

The scapular stabilisers’ balanced actions function as force couples for the scapula (Cools et al. 2004; Inman et al. 1944; Ludewig & Reynolds 2009; Vanderstukken et al. 2020). The upper force couple consists of the upper fibres of the serratus anterior and the upper trapezius muscles (Bagg & Forrest 1986). The lower force couple, which consists of the lower fibres of the serratus anterior and the lower trapezius, also plays a vital role in the efficient functioning and control of scapulohumeral movement (Inman et al. 1944). Force couples can be defined as ‘two equal forces acting in opposite directions to rotate a part about its own axis of movement’ (Kent 1971:870). The strength of the individual scapular-stabilising muscles is important, but the ideal force ratio within the different force couples is of more significance (Bagg & Forrest 1988; Kibler 1998a).

A muscle tested in isolation, without taking synergistic or antagonistic muscle actions into consideration, may test strongly in a normal manual muscle testing position, but may perform poorly in a functional movement activity (Magarey & Jones 2003). The strength of muscles tested in isolation is often considered less important than the muscle balance within the muscle force couples (Kibler 1998b; Kibler & Chandler 1994). In a clinical setting, visual observation, as well as testing of the periscapular muscles, is warranted in the evaluation of scapular dysfunction (Kibler et al. 2013).

Optimal positioning of the glenoid, mainly by the scapular stabilising muscles, is a key component in the ideal functioning of these stabilising muscles. The ideal positioning of the glenoid leads to optimum glenohumeral arthrokinematics, thereby increasing upper limb mobility (Mottram 1997). Labriola et al.’s (2004) comment is relevant in this instance:

[*I*]mproved understanding of the contributions of muscle forces, not only towards joint stability but also towards instability, will improve rehabilitation protocols in the treatment of joint stability throughout the body. (p. 802)

The synergistic action of serratus anterior lower fibres and the lower trapezius is important for the control of the upward rotation of the scapula, in both the sagittal and the frontal plane of elevation (Bagg & Forrest 1986; Johnson et al. 1994). The disruption of this force couple, in which the lower trapezius and the serratus anterior lower fibres play a significant role, could have a negative knock-on effect on the upward rotation of the scapula (Ebaugh, McClure & Karduna 2005; Johnson et al. 1994; Phadke, Camargo & Ludewig 2009).

Antagonistic and synergistic action of the scapulothoracic muscles is required, in varying degrees, for efficient stability and movement of the scapula during glenohumeral elevation (Lucado 2011). Even though our study did not look at the scapulohumeral movement, the scapular stabilisers and, particularly, the ideal force couple ratios of the scapular stabilisers are needed for the execution of the scapulothoracic motion. Struyf et al. (2014) concluded that knowledge of the scapulothoracic motion is important for several reasons: (1) for the development of clinical assessment tests that can flow from it; (2) for preventative strategies that can be developed for asymptomatic individuals; and (3) for developing effective treatment strategies for symptomatic patients.

The objective of our study was thus to determine typical values for the force couple ratios within the different force couples of the scapula’s stabilising musculature in pain-free shoulders in the nondominant versus the dominant arms of both sexes. As full an understanding as possible of the normal relationship that exists within the two sides of the body is needed. Strength differences determined between sides can be utilised by clinicians to develop practical treatment strategies (Gulick et al. 2001; Michener et al. 2016). Increased upper body strength differences are noted in studies of women and men, with increased strength observed in men (Bailey et al. 2015; Bartolomei et al. 2021; Miller, MacDougall & Tarnopolsky 1993). In a time where the call for evidence is practice-based, there is a need for better scientific protocols. Therefore, establishing typical values for the force production ratios of the scapular stabilisers can contribute towards an improved understanding of the shoulder complex.

## Methods

This was a cross-sectional, quantitative study. Data collection took place in the University of the Witwatersrand research movement laboratory. Participants were recruited from the Department of Physiotherapy at the associated tertiary institution and from the general public (schools, church groups and sport clubs). Participants between 18 and 35 years of age were chosen because, in this age group, underlying musculoskeletal dysfunction is least likely to be present (Djade et al. 2020; Monrad et al. 2018). Both female and male participants without shoulder pain were included. Participants who had had previous shoulder surgery and/or with underlying cervical dysfunction were excluded. Data collection took place between 27 June 2016 and 18 March 2017.

To determine the sample size, a Pearson correlation coefficient was calculated to determine the correlation between the variables of the upper trapezius and the lower trapezius using existing data, which were collected from 354 patients (Schober, Boer & Schwarte 2018). Our own data pool of 354 evaluated patients was utilised for the sample size calculation. It was necessary to use these data because, at the time of our study, no other data for comparison existed in the literature. The effect size used was 0.3449. Descriptive statistics were used, with the means and standard deviations of the upper trapezius and lower trapezius muscles as the variables. Once the correlation coefficient was calculated (*r* = 0.7), an *a priori* sample size was calculated using G*Power (version 3.1.9.2). Using an alpha of 5%, a power of 95% and a two-tailed design, it was determined that the sample size required under these conditions would be *n* = 58.

### Instrumentation

The muscle strength of the scapular muscles was measured in kilogram force (kgf) using a manual handheld dynamometer (Health Industries Inc., West Jordan, UT, USA). This comprised the following force couples: upper trapezius versus lower; middle trapezius versus serratus anterior upper fibres; serratus anterior lower fibres versus rhomboids; serratus anterior lower fibres versus lower trapezius and lower trapezius versus rhomboids, in both the shoulders of the participants.

### Procedure

**FIGURE 1:**
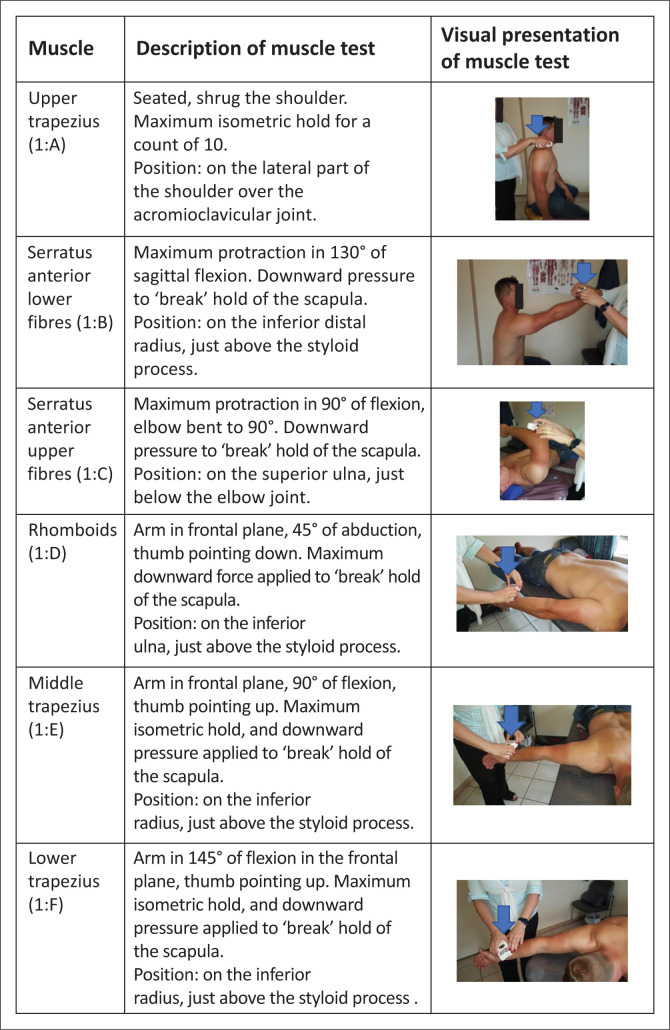
Muscle descriptions and testing positions used (*n* = 58).

Force measurements (muscle strength) were obtained, and each muscle was tested twice and in the same order each time. The following sequence of testing positions was followed: upper trapezius (Figure 1a), then serratus anterior lower fibres (Figure 1b), followed by serratus anterior upper fibres (Figure 1c), rhomboids (Figure 1d), middle trapezius (Figure 1e) and lower trapezius (Figure 1f). Testing positions used for the upper trapezius, lower trapezius and serratus anterior were based on the descriptions of Hislop and Montgomery (1995). For the middle trapezius and rhomboids, the muscle testing techniques of Kendall and McCreary (1993) were used. To calibrate the handheld dynamometer, it was held down on an electronic domestic scale before each testing session. The ‘break’ principle of the maximum force measurement test was used (Hislop & Montgomery 1995). That position was then held for a count of 10 s. The better result from the two tests was used for data analysis.

The following factors determined the choice of the muscle pairs of the force couples: In the literature, the upper force couple is said to consist of the upper trapezius and the serratus anterior upper fibres (Bagg & Forrest 1986; Schenkman & Rugo de Cartaya 1987). In our study this was changed by coupling the upper trapezius with the lower trapezius because the upper trapezius is active mainly in elevation of the scapula rather than upward rotation of the scapula (Fey et al. 2007; Johnson et al. 1994), and the lower trapezius is active in depression of the scapula (Johnson et al. 1994). The serratus anterior lower fibres and the lower trapezius are seen by many as the only true upward rotators of the scapula (Ekstrom, et al. 2004; Phadke, et al. 2009).

Serratus anterior upper fibres were coupled with the middle trapezius in our study. Johnson et al. (1994) found that the middle trapezius is more active in retraction of the scapula, and Ekstrom et al. (2004) concluded that the serratus anterior upper fibres are more involved in protraction of the scapula. These were the deciding factors for the proposed force couple of the middle trapezius versus serratus anterior upper fibres. We decided to divide the lower force couple of serratus anterior lower fibres and the lower trapezius further into force couples: between the serratus anterior lower fibres and the lower trapezius, between the rhomboids and the lower trapezius and between the rhomboids and the lower trapezius.

This decision was reached because differences have been demonstrated in the electromyographical (EMG) activity of serratus anterior lower fibres in abduction, compared to flexion, and for the lower trapezius versus serratus anterior lower fibres in flexion (Basmajian & De Luca 1985).

### Statistical analysis

We utilised descriptive analysis. The means, standard deviations and ranges were calculated for all force measurements. The Shapiro–Wilk test was used to determine the normality of the data (Mishra et al. 2019). The data presented a Gaussian distribution and paired t-tests were employed for the comparison between the nondominant and the dominant sides; independent t-tests were utilised for the comparison between the female and the male participants. Graph Pad 5 (Prism, San Diego, USA) was used for the statistical analysis. Statistical significance was set at 5%.

### Ethical considerations

Prior to their participation in our study, written informed consent to take part in our study was obtained from all participants. Written informed consent to use the photographs was also obtained. Ethical clearance to conduct this study was obtained from the Human Research Ethics Committee (Medical) of the University of the Witwatersrand, reference number: M160515.

## Results

### Participants

A total number of 58 participants took part in the force measurement data collection sessions (Table 1). An equal number of women and men participated.

**TABLE 1:** Demographic and anthropometric information (*n* = 58).

Variables	Combined group		Female		Male	
	x	SD	x	SD	x	SD
Age (years)	25.4	4.6	24.9	4.7	25.9	4.7
Mass (kg)	80.2	25.1	69.0	11.9	91.1	29.6
Height (cm)	171.6	10.3	165.0	6.6	178.0	9.3
Arm dominance (L:R)	5:53		4:25		5:53	

x, mean; SD, standard deviation; L, left; R, right.

The mean force values of the male participants were higher than those of the female participants for all comparisons (*p* < 0.001) (Table 2). No difference was observed between the nondominant and dominant sides for any of the muscles within the female or male groups (Table 2).

**TABLE 2:** A comparison between the force measurements in female (*n* = 29) and male participants (*n* = 29) (*n* = 58) (measurements in kgf: means and SD).

Muscles	Female				Male				
	Minimum	Mean	SD	Maximum	Minimum	Mean	SD	Maximum	*p*
**Nondominant**									
**UT**	7.60	9.75	1.06	12.60	9.10	11.85	1.74	16.40	< 0.001
**MT**	1.80	2.85	0.78	4.70	2.90	4.92	1.49	6.80	< 0.001
**lt**	1.80	2.85	0.65	4.10	2.90	4.34	0.97	6.80	< 0.001
**sau**	5.40	8.10	1.51	11.10	6.90	10.98	2.09	16.60	< 0.001
**sal**	3.50	5.39	1.28	9.50	5.80	9.31	1.98	13.10	< 0.001
**rh**	2.50	3.74	0.88	6.20	4.10	6.63	1.84	11.70	< 0.001
**Dominant**									
**UT**	7.40	9.53	1.20	12.70	9.50	11.18	1.32	15.80	< 0.001
**MT**	2.00	3.05	0.74	4.50	2.60	4.98	1.55	9.70	< 0.001
**LT**	1.50	2.76	0.59	3.60	2.70	4.44	1.38	8.80	< 0.001
**SAU**	4.80	7.80	1.51	10.30	7.30	10.56	1.47	13.40	< 0.001
**SAL**	3.50	5.33	1.04	8.10	6.30	9.15	1.71	12.20	< 0.001
**RH**	2.10	3.85	0.91	6.30	3.50	6.88	2.31	15.00	< 0.001

UT, upper trapezius; LT, lower trapezius; MT, middle trapezius; SAU, serratus anterior upper fibres; SAL, serratus anterior lower fibres; RH, rhomboids; SD, standard deviation; kgf, kilogram force.

Specific force couple ratios were calculated in all the force couples in both female and male participants. The mean force couple ratio for upper trapezius versus lower trapezius was found to be higher in the nondominant side, as well as the dominant side, in the women, compared to the men (*p* < 0.001) (Table 3). The ratio between the middle trapezius versus serratus anterior upper fibres in both nondominant and dominant sides of the women was lower compared to that of the men (*p* < 0.001) (Table 3). No differences were observed in the ratios between the nondominant and the dominant sides of the men (Table 3).

**TABLE 3:** A comparison between the force couple ratio measurements in male (*n* = 29) and female participants (*n* = 29) (*n* = 58).

Muscle ratios	Female				Male				
	Minimum	Mean	SD	Maximum	Minimum	Mean	SD	Maximum	p
**Nondominant**									
**UT:LT**	2.24	3.97	0.88	5.20	1.66	2.85	0.73	4.42	< 0.0001*
**SAL:LT**	1.21	2.15	0.57	4.52	1.22	2.21	0.53	3.54	0.6808
**MT:SAU**	0.23	0.35	0.08	0.54	0.27	0.45	0.08	0.60	< 0.0001*
**SAL:RH**	1.12	1.48	0.42	3.39	0.88	1.45	0.26	1.90	0.7353
**LT:RH**	0.48	0.70	0.16	1.00	0.36	0.69	0.19	1.07	0.6934
**Dominant**									
**UT:LT**	2.34	3.63	0.97	6.00	1.57	2.70	0.72	4.15	< 0.0001*
**SAL:LT**	1.46	1.97	0.27	2.63	1.39	2.15	0.45	3.29	0.0627
**MT:SAU**	0.23	0.40	0.10	0.52	0.25	0.47	0.12	0.72	0.0135*
**SAL:RH**	1.09	1.41	0.21	1.87	0.81	1.40	0.31	2.17	0.8923
**LT:RH**	0.42	0.74	0.17	1.10	0.30	0.17	0.6	1.02	0.1073

UT, upper trapezius; LT, lower trapezius; MT, middle trapezius; SAU, serratus anterior upper fibres; SAL, serratus anterior lower fibres; RH, rhomboids; SD, standard deviation.

Significant differences are denoted by an *.

## Discussion

Functionally, the muscle balance within force couples, as well as the strength of individual muscles and muscle groups, should be considered. It is in our interest as clinicians to establish typical values for muscle strength. The aim of our study was thus to determine the ratios of the force couples between and within both shoulders, in both sexes, in the following force couples: upper trapezius versus lower trapezius; serratus anterior upper fibres versus middle trapezius; serratus anterior lower fibres versus lower trapezius; serratus anterior lower fibres versus rhomboids; and lower trapezius versus rhomboids.

Definitive force couple ratios were found to exist in all the scapular stabilisers in both sexes. Margery and Jones (2003) alluded to the fact that testing a muscle out of context could be misleading. This is particularly true of the scapular stabilisers, where it can be deceptive if only the individual muscles are tested and the force couple ratios are ignored. We showed that, even though higher mean force values existed in all the individual forces between the sexes, only certain force couple ratios were found to be different. Because our study was conducted on pain-free shoulders, it can be seen as supporting Kibler and McMullen’s (2003) statement that scapular asymmetry can exist in shoulders without musculoskeletal dysfunction. One of the proposals of our study is the hypothesis of a different resting position of the scapula in men compared to women. The position was not directly measured. This was concluded because of a significant difference in some of the proposed force couple ratios between the female and male sample groups, as well as the increased mean force values in the individual values of the upper trapezius and the serratus anterior upper fibres in men, as indicated in the results section.

Large differences were found between the female and the male sample groups in the mean force couple ratios of the upper trapezius versus the lower trapezius and the serratus anterior upper fibres versus the middle trapezius. However, these observed differences in the force couple ratios do not indicate the presence of dyskinesis (or dyskinesia). As the differences in the force couple ratios arrived at here were measured and observed in healthy individuals with normal shoulders, these ratios could indicate the norm in healthy shoulders. A disruption of the concluded force couples would therefore be required to cause dyskinesis (or dyskinesia). In other words, dyskinesia or dyskinesis would follow only if an imbalance is created between the muscles paired in a particular force couple. Hence, visual asymmetry cannot be an indication of musculoskeletal dysfunction. Only if the force couple ratios are measured and compared to the data collected here might underlying dysfunction be suspected. The suggestion by Cools et al. (2003) that scapular asymmetry might not solely emanate from underlying musculoskeletal dysfunction, unless proven by more tests than just visual observation, could confirm the statement proposed in our study.

The force couple ratio of serratus anterior lower fibres versus lower trapezius is of particular importance, as it controls the inferior-medial border of the scapula (Hébert et al. 2002). It follows that prominence of the inferior-medial corner of the scapula could indicate disruption of this force couple (Hébert et al. 2002). The serratus anterior lower fibres and lower trapezius are also the muscles most frequently weakened and compromised in painful shoulders (Solem-Bertoft, Thomas & Westerberg 1993). The upward rotation of the scapula by the serratus anterior lower fibres is counteracted by the synchronous activity of the lower trapezius (Perry 1978). The serratus anterior lower fibre is the main muscle involved in the posterior tilt action of the scapula (Ekstrom et al. 2004). It was an interesting finding that no real differences existed in the force couple ratios of serratus anterior lower fibres versus lower trapezius, within and between the dominant and the nondominant sides in both sexes, even though the individual male strength values were much higher than those of the female group.

External rotation (or posterior tilt) of the scapula occurs at the end of the range of humeral elevation (Ludewig & Reynolds 2009; McClure et al. 2001). Subacromial impingement frequently occurs when the shoulder is flexed or abducted in the midrange of movement. In the final stages of elevation of the scapula (120° and more), the main muscles contributing to upward rotation are the lower trapezius and the serratus anterior lower fibres (Bagg & Forrest 1986). The biomechanical importance of this particular couple ratio (serratus anterior lower fibres and lower trapezius) has been clearly identified in the literature. The importance of not just testing the isolated scapular stabilisers but also paying closer attention to the synergistic and/or antagonistic force couple ratios, as we determined supports the statement by Magarey and Jones (2003) that a muscle must not be tested in isolation. The typical values of the force couple ratios of serratus anterior lower fibres and lower trapezius can therefore serve as a guideline in the early identification and correction of imbalances of this synergistic force couple. Potential subacromial impingement can theoretically be prevented.

The goal of a shoulder examination is the identification of scapular asymmetry in symptomatic patients. There is, however, still uncertainty about the meaning of scapular asymmetry when it is present during the physical examination. More research is needed in this area to clarify the presence of scapular asymmetry (Kibler & McMullen 2003). This, in turn, will help develop treatment plans for symptomatic individuals (Ludewig & Reynolds 2009). Scapular asymmetry can thus not be seen as conclusive evidence of an underlying pathology, unless this is apparent from more tests than just visual observation (Cools et al. 2003). If visual scapular asymmetry can be linked to the disruption of the force couple ratios we arrived at in our study, more clarification of the meaning of asymmetry present in the symptomatic patient can be provided.

The determination of typical values for the different force couple ratios of the scapular stabilisers can add more data to the evaluation of scapular kinematics. The scapula is stabilised, both statically and dynamically, by the scapular stabilising muscles. Therefore, it is important to know the values of the force couple ratios for rehabilitation and evaluation purposes. Clinicians can use the force couple ratios presented here as a guide for rehabilitation. The simultaneous action of two opposing muscular force couples is considered important for the effective functioning of that particular joint motion. This is especially true regarding scapular stability during glenohumeral movement (Oatis 2009; Schory et al. 2016). It is therefore not only the isolated muscle strength of the scapular stabiliser muscles that is important but also the ratios in the synergistic and antagonistic force couples of the scapular stabilisers that function to control the complex actions of the scapula. The force couple ratios we calculated serve as a reminder of the importance thereof.

A limitation to our study is that, although the sample size used in our study was large enough to add sufficient credence to our study and to the inferential statistics used, using a larger sample from a healthy population would add to the database of typical figures for the scapula. The data collected could be used as a reference point for patients in the same age group, 19–35 years of age, but cannot necessarily be applied to the general population.

## Conclusion

In a time where the call is for more objective, sensitive and measurable evaluation methods, the results expressed here could aid in creating a database for force measurements of the scapular muscles. The benefit of knowing the values calculated in our study of the scapular stabilisers lies in their application in clinical practice.
